# A Deep-Hole Microdrilling Study of Pure Magnesium for Biomedical Applications

**DOI:** 10.3390/mi14010132

**Published:** 2023-01-03

**Authors:** Margherita Pizzi, Francesco De Gaetano, Marco Ferroni, Federica Boschetti, Massimiliano Annoni

**Affiliations:** 1 Department of Mechanical Engineering, Politecnico di Milano, 20156 Milan, Italy; 2 Department of Chemistry, Materials and Chemical Engineering “Giulio Natta”, Politecnico di Milano, 20133 Milan, Italy; 3 MgShell S.r.l., 20133 Milan, Italy

**Keywords:** microdrilling, magnesium, microholes, micromachinability, holes quality, chip formation, chip thickness, biomedical device

## Abstract

The mechanisms of deep-hole microdrilling of pure Mg material were experimentally studied in order to find a suitable setup for a novel intraocular drug delivery device prototyping. Microdrilling tests were performed with 0.20 mm and 0.35 mm microdrills, using a full factorial design in which cutting speed $v_{c}$ and feed $f_{z}$ were varied over two levels. In a preliminary phase, the chip shape was evaluated for low feeds per tooth down to 1 μm, to verify that the chosen parameters were appropriate for machining. Subsequently, microdrilling experiments were carried out, in which diameter, burr height and surface roughness of the drilled holes were examined. The results showed that the burr height is not uniform along the circumference of the holes. In particular, the maximum burr height increases with higher cutting speed, due to the thermal effect that plasticizes Mg. Hole entrance diameters are larger than the nominal tool diameters due to tool runout, and their values are higher for high $v_{c}$ and $f_{z}$. In addition, the roughness of the inner surface of the holes increases as $f_{z}$ increases.

## 1. Introduction

Nowadays, magnesium (Mg) and Mg-based alloys play a very interesting role in the field of biomaterials [1]. As one of the essential elements for the regulatory functions of the human body [2,3], the use of magnesium for biomedical devices is therefore desirable. Due to its absorbable nature in biological fluids, the biocompatibility of its corrosion products and its low toxicity, this material represents an optimal candidate for several applications, especially where an implantable device is required to perform its function for a limited period of time and a short-term integrity is necessary [4,5]. The corrosion inside body fluids allows the device to disappear completely once it is no longer useful avoiding removal surgeries [6,7].

In this specific context, a Mg-based drug delivery device for the treatment of age-related macular degeneration (AMD), currently treated with monthly injections of anti-vascular endothelial growth factors (anti-VEGF) drugs in the posterior chamber of the eye [8], might be a solution to be proposed. The high frequency of injections not only causes various complications, such as endophthalmitis, but also leads patients, caregivers and physicians to bear a high overall care burden. This fact can be significant and can lead to non-compliance or complete discontinuation of treatment [9]. With this novel application, the main goal is to avoid these issues by reducing the number of injections while achieving the same results. The behaviour of the device was investigated in previous studies by means of a numerical model for a critical evaluation of the effective shear stress field induced by ocular fluid dynamics on its free external surfaces. This approach showed the possibility of achieving uniform controlled corrosion of the device [10,11,12].

Currently, the manufacturing technology suitable for the prototyping of this innovative Mg-based drug delivery system needs to be investigated. In particular, the critical aspect in its realisation is closely linked to the presence of cavities, which provide the drug housing with a very high depth-to-diameter ratio.

In the field of manufacturing, a wide range of possible technologies and machining strategies to transform biocompatible materials into biomedical implants can be found. In particular, two methods can be defined: conventional and unconventional. The first includes milling, turning and drilling, while the second includes abrasive water jet machining (AWJM), ultrasonic machining (USM), ion beam machining (IBM), laser beam machining (LBM), electric discharge machining (EDM) and electron beam machining (EBM) [13]. Among these technologies, mechanical microdrilling has the advantage of achieving good geometrical quality and at the same time good productivity. For this reason, this specific study focused on micromechanical cutting methods. These are commonly used to obtain the final shape of magnesium-based devices, but very few studies can be found in the literature [14,15]. Moreover, in microscale machining, a number of issues that are fundamentally different from those of macroscale machining emerge. These affect the basic mechanisms of the process. The consequences lead to changes in the chip formation process, cutting forces, vibrations and process stability, as well as the resulting machined surface [16].

One of the biggest obstacles in this field is the minimum chip thickness effect. When working in the micro area, the chip volume decreases, leading to an uncut chip thickness h that can be compared to the size of the tool radius $r_{e}$. The relationship between these two factors determines the material cutting mechanism. It can be observed in the literature that if the uncut chip thickness h is less than a critical value called minimum chip thickness $h_{m}$ dependent on the cutting edge geometry, depth of cut, feed rate and target material, chip formation will not be present. In fact, what happens is that the workpiece material is subjected to ploughing due to elastic-plastic deformation [16,17,18].

Another aspect to consider, especially in deep microdrilling, is the proper evacuation of chips. Indeed, when holes with a high depth-to-diameter ratio have to be drilled, the amount of chip formed during the process is high. Therefore, if chips are not removed correctly, they can clog the flute, bringing to high values of forces on the drill, which in turn raises the temperature. These phenomena reduce hole quality and accelerate tool wear and breakage [19]. To overcome this problem, peck drilling is often used. In fact, this strategy is a commonly used method to minimise problems related to excessive cutting forces and torque in deep microdrilling [20,21]. It consists of drilling the microhole with an intermittent feed, so that the micro drill alternates phases in which it cuts (peck phases), and phases in which it does not cut the material at all.

There are numerous studies in the literature concerning the drilling process, but there are few cases in which the micromachining of pure magnesium and its alloys is considered.

Some experimental micromilling tests were conducted to investigate the micromachinability of Mg_5_Sn_4_Zn alloy (TZ54) as a biomedical material compared to pure Mg [14]. By studying the variation in cutting forces, surface quality, and burr width, appropriate cutting parameters were defined. It has been observed that the surface quality decreases and burr formation increases with a feed per tooth lower than the minimum chip thickness. Furthermore, an experimental study was conducted on the micro drilling mechanisms of a Mg metal matrix composite and pure Mg. The influence of drilling parameters such as rotational speed and feed rate on the morphology of the hole surface was studied [15]. The results showed that smaller feed and rotation speeds are used in microdrilling to achieve lower burr heights. In this particular study, the minimum chip thickness for Mg has been measured equal to 1.7 μm.

In this paper, the mechanisms of deep-hole microdrilling of pure Mg were experimentally studied in order to find a suitable setup for a novel intraocular drug delivery device prototyping. For this reason, a feasibility study of deep-hole microdrilling of pure magnesium was conducted in order to understand the suitability of this technology for the future manufacture of the device. Microdrilling tests were performed with 0.20 mm and 0.35 mm microdrills, compatible with the device cross-sectional dimensions using a full factorial design in which cutting speed $v_{c}$ and feed $f_{z}$ were varied over two levels. Chip formation for low feed values down to 1 μm was investigated to confirm the choice of working cutting parameters. Therefore, entrance diameters and burr height were examined. Moreover, the surface roughness of the inner hole wall was measured.

## 2. Materials and Methods

Microdrilling tests were carried out on four circular pure magnesium specimens (diameter *D* = 20 mm, thickness *th* = 10 mm). Table 1 shows the material properties. Hardness was measured using a microhardness tester (FM-810, FUTURE-TECH CORP., Kawasaki, Japan), while density was measured using an analytical balance (MC-1 Analytic AC210P, Sartorius AG, Göttingen, Germany) with a density determination kit (YDK 01, Sartorius AG, Germany).

**Figure 1:**
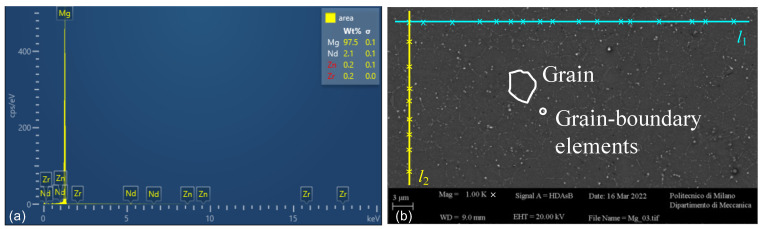
Metallographic analysis of the Mg sample. (**a**) Composition of the sample. (**b**) SEM grain size, grain-boundary elements, $l_{1}$ and $l_{2}$ used for grain size.

When the thickness of the uncut chip is of the same order of magnitude as the grain size of the material, the workpiece material can no longer be assumed to be homogeneous and isotropic [22]. For this reason, a metallographic analysis of the bulk material was performed before the tests using a field emission scanning electron microscope (FE-SEMs, ZEISS Sigma 500 Gemini, Carl Zeiss, Oberkochen, Germany). In Figure 1 the composition of the material and the grain boundaries are shown. White spots can be observed: these are grain-boundary elements as neodymium and zinc. These analyses revealed that the material, previously studied for biomedical applications associated with magnesium [23,24], contains very low amounts of alloying elements. This makes such material suitable for the present application, therefore it will conventionally be treated as pure Mg.

Three horizontal lines of length $l_{1}$ = 55 μm and three vertical lines of length $l_{2}$ = 35 μm were drawn on the micrograph (see Figure 1b) in order to measure the average size of the grain $\bar{d}$. Dividing $l_{1}$ and $l_{2}$ by the number of intersections with the grain boundaries, 6 values of $\bar{d}$ were obtained. The average grain size of the workpiece material was evaluated as 3 μm (σ = 0.27 μm).

### 2.1. Cutting Tools and Drilling Strategy

**Figure 2:**
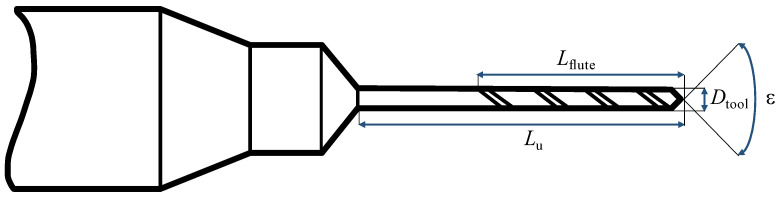
Microtwist drills geometry.

**Figure 3:**
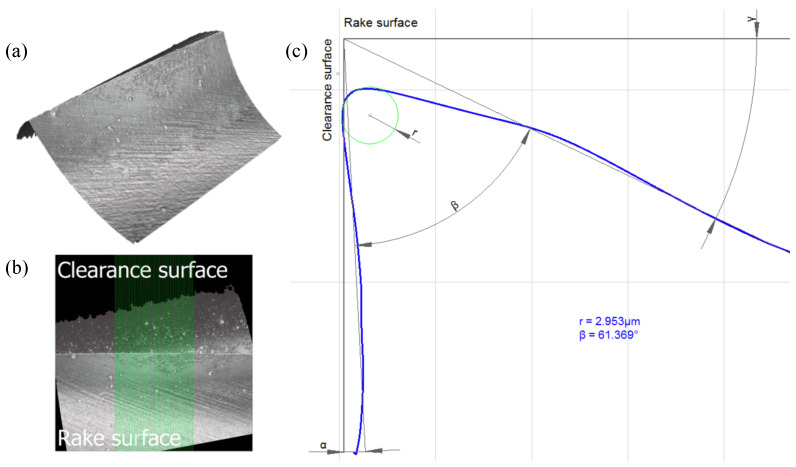
Cutting edge radius measurement by means of Alicona InfiniteFocus G5plus. (**a**) Acquisition of the cutting edge with the EdgeMasterModule. (**b**) Portion of the cutting edge highlighted in green containing the 800 profiles to be averaged. (**c**) Averaged profile of the cutting edge evaluated along the region of interest.

A 0.20 mm (Custom, Louis Belét, Vendlincourt, Switzerland) and a 0.35 mm (2.CD. 080035.IN, Mikron Tool, Agno, Switzerland) coated microtwist drills with two cutting edges were used for the experimental campaign (see Figure 2 and Table 2). The radius of the cutting edges $r_{e}$ for both the microdrills was measured using the 3D microscope Alicona InfiniteFocus G5plus (Bruker Alicona, Graz, Austria). This value was measured using the appropriate EdgeMasterModule, which directly quotes the geometrical features of the tool (see Figure 3). The software algorithm, with default settings considers 50% of the length of the acquired cutting edge, and measures 800 equidistant profiles (green band in Figure 3b). This means that $r_{e}$, which corresponds to the radius of the circle that best fits the points of the profile, is averaged over 800 profiles.

The Kern EVO high-precision machining center was used for the experimental campaign. Its spindle can rotate at speeds ranging from 500 to 50,000 rpm and can provide a maximum power of 6.4 kW. The machine accuracy on the workpiece is declared to be ±2.0 μm.

**Figure 4:**
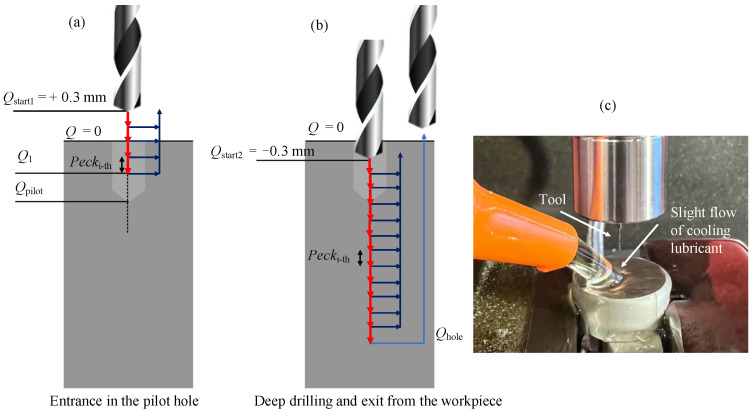
Peck drilling strategy. (**a**) The peck of the tool starts outside the workpiece with a partial retraction inside the pilot hole until $Q_{1}$. (**b**) After entering the pilot hole, the microdrill performs the same strategy remaining inside the hole, rising to the rising point $Q_{\mathrm{start}2}$ after each peck. (**c**) Setup of the drilling process, in which the lubricant is supplied at a slight flow rate.

A centering operation was performed in order to achieve a better stability of the microdrill during the deep-drilling operation. In this case a Louis Belét (342d0.21, *D*_pilot0.21_ = 0.21 mm) and a MikronTool (2.PFS.035.1, *D*_pilot0.35_ = 0.35 mm) pilot drills were used to obtain a pilot hole depth *Q*_pilot_. It was preferred to use a pilot drill with a diameter 0.01 mm larger than the 0.20 mm microdrill in order to prevent tool breakage due to runout effects. The parameters $v_{c}$ = 23 m/min – $f_{z}$ = 5 μm and $v_{c}$ = 50 m/min – $f_{z}$ = 5 μm were used for the 0.21 mm and 0.35 mm pilot holes, respectively. Then, a deep drilling strategy with partial retraction of the tool was performed. Figure 4a,b explain the adopted solution. The tool in step (a) enters the pilot hole using the peck drilling strategy, in which at each peck rises to the rising point $Q_{\mathrm{start}1}$ outside the workpiece, until it reaches the safety depth $Q_{1}$. The value of $Q_{1}$ was chosen in order to get the tool into the pilot hole as far as possible, but without colliding with the surface of the bottom of the pilot hole, and thus avoid tool breakage. This quote represents the point at which the entrance into the pilot hole ends to start deep drilling, and its value changes depending on the pilot hole. In fact, 0.20 mm and 0.35 mm holes have pilot holes with different depths, so consequently $Q_{1}$ also changes. Specifically, $Q_{1}$ is equal to −0.48 mm and −0.57 mm for the 0.20 mm and 0.35 mm holes respectively. Subsequently, in step (b) the tool rises to $Q_{\mathrm{start}2}$ inside the workpiece in order to achieve a better tool stability during pecking, from which the actual deep-drilling begins: at each peck the tool goes to the rising point $Q_{\mathrm{start}2}$ until the final depth of the hole $Q_{\mathrm{hole}}$ is obtained.

The used cutting conditions are reported in Table 3. The strategy was carefully chosen to promote heat dissipation, avoid chip adhesion and facilitate chip evacuation. Infact, the cooling lubricant Blasogrind HC5 was used during the drilling operations in order to prevent all the issues generally encountered in deep microdrilling [25]. The lubricant was supplied during machining with a very slight flow in order not to deflect the tool, as shown in Figure 4c.

Moreover, in this experimental study the peck values were chosen in order to break the chip frequently, and thus reduce its length for better evacuation. This technique, with the rising of the tool in these holes with high aspect ratios, contributed greatly to the success of the hole and to prevent the drill from breaking.

**Figure 5:**
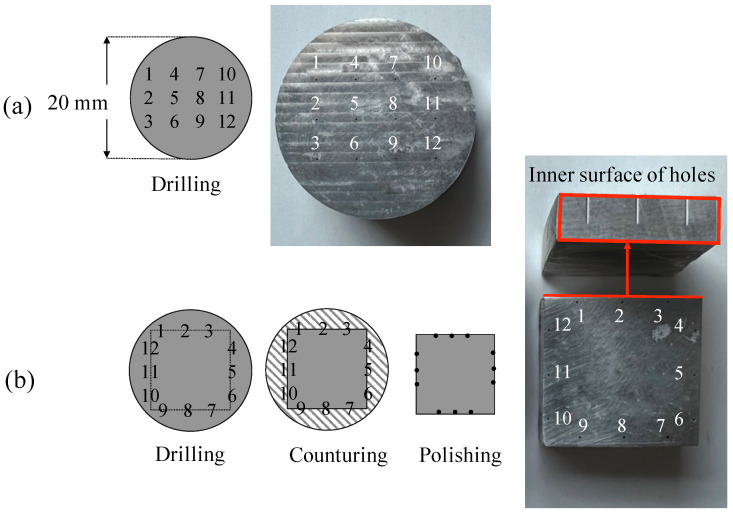
Mg drilled samples. (**a**) Sample with 12 holes for entrance diameters and burr heights measurements. (**b**) Sample with 12 holes for the measurements of roughness of the inner surface of the holes.

In this experimental campaign, two Mg samples were drilled for both 0.2 mm and 0.35 mm holes: one for measuring diameters and burr height, the other for measuring the internal roughness of the holes (see Figure 5). A 2^2^ factorial design with 3 replicates (12 holes) was selected for the experimentation, thus varying the $f_{z}$ and $v_{c}$ factors on 2 levels (see Table 4). The range of values for feeds and cutting speeds were selected in agreement with the manufacturers of the respective tools. For these measurements, an Alicona InfiniteFocus G4 (Bruker Alicona, Austria) was implied.

### 2.2. Chip Analysis

The cutting parameters were chosen according to the range recommended by the respective manufacturers. In addition, a qualitative chip analysis was performed to be sure to work in cutting and not ploughing conditions.

If $h_{m}$ is assumed to be between 20–40% of the cutting edge radius for Mg as in the case of aluminum alloys [26,27,28], $h_{m}$ assumes a maximum value equal to 1.81 μm and 1.18 μm for the 0.35 mm and 0.20 mm holes respectively, considering the value of $r_{e}$ in Table 2. The chip thickness for the 0.35 mm and 0.20 mm drills $h_{0.35}$ and $h_{0.20}$ can be determined using Equation (1).

(1) $$h=f_{z}\cdot sin\left(\epsilon /2\right)$$

Substituting $f_{z}$ and ϵ into the Equation (1) with the values given in Table 2 and Table 4, a value of h greater than $h_{m}$ is always obtained:

- $h_{0.20}$ = 2.17 μm for $f_{z}$ = 2.5 μm
- $h_{0.20}$ = 4.33 μm for $f_{z}$ = 5 μm.
- $h_{0.35}$ = 4.53 μm for $f_{z}$ = 5 μm
- $h_{0.35}$ = 13.59 μm for $f_{z}$ = 15 μm

As a result, it can be stated that the material removal should take place through shearing and not ploughing of the target material.

In order to verify this condition, tests were performed to observe the chip formation as the feed rate decreased to 1 μm. Then, 3 holes were drilled at different $f_{z}$ values, as shown in the Table 5, for the 0.20 mm and 0.35 mm drill. In this case, the cutting speed was kept fixed, since it should not play a significant role in chip breakage [29].

The holes were drilled following the strategy described above (Figure 4 and Table 3), changing only the hole depth, which was kept at 2 mm. In addition, no lubricant was used, in order to be able to collect the chips more easily. Then, the collected chip morphology was observed with the scanning electron microscope ZEISS EVO 50 (Carl Zeiss, Oberkochen, Germany). The chip thickness was measured with the software ImageJ for $f_{z,a}$ and $f_{z,c}$ and compared with the calculated theoretical values h using the Equation (1) (see Table 6).

### 2.3. Hole Quality

For the study of hole quality, data on maximum burr height, hole entry diameter and internal roughness were collected for each hole.

#### 2.3.1. Burr Height Measurement

**Figure 6:**
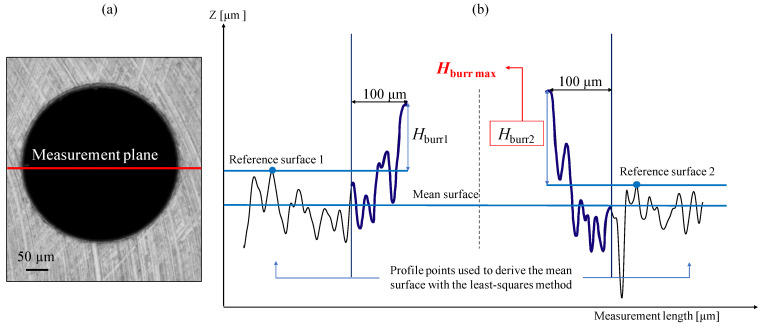
Burrs height measurements. (**a**) Measurement plane selected. (**b**) $H_{\mathrm{burr}}$ profile and mesurement procedure.

BS EN ISO 8785:1999 [30] was followed for the burr height measurement. With the Alicona software, using the ’ProfileFormMeasurement’ function, a line was drawn through the centre of the hole, as shown in Figure 6a. This allowed the profile shape of the hole belonging to the section plane passing through the axis of the hole to be obtained (Figure 6b. Initially, the main surface was defined. Then, for each side of the profile, the reference surface was also set, parallel to the main surface and passing through the maximum peak considered 100 μm away from the highest point of the burr. In this way the burr height for each side was measured as the distance between the corresponding reference surface and the maximum peak of the burr itself. For each hole, two values of burr height $H_{\mathrm{burr}1}$ and $H_{\mathrm{burr}2}$ were taken and the greater of the two was considered and named $H_{\mathrm{burr}\max}$. The distances between the peaks and the reference surfaces were measured using the ‘Height Step–Maximum distance’ tool. This tool allows the distance measurement mode, with which the areas that define the reference level and the measurement level can be set, thus obtaining the height step (maximum distance).

#### 2.3.2. Diameter Measurements

**Figure 7:**
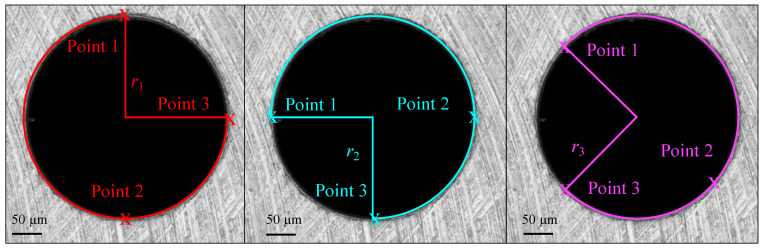
Diameters measurements for each hole: selected points for the three best fitted circles.

The diameter of the holes was measured by using the Alicona software, with a manual drawing tool in the 2DImageMeasurements. By selecting three points on the circumference of the holes, a circle that exactly intersects the three points was drawn. This function returns the radius of that circle. For each hole, the best-fitting 3-point circle has been drawn three times in order to obtain a mean value for each radius. The points were selected as shown in Figure 7.

#### 2.3.3. Roughness Measurements

**Figure 8:**
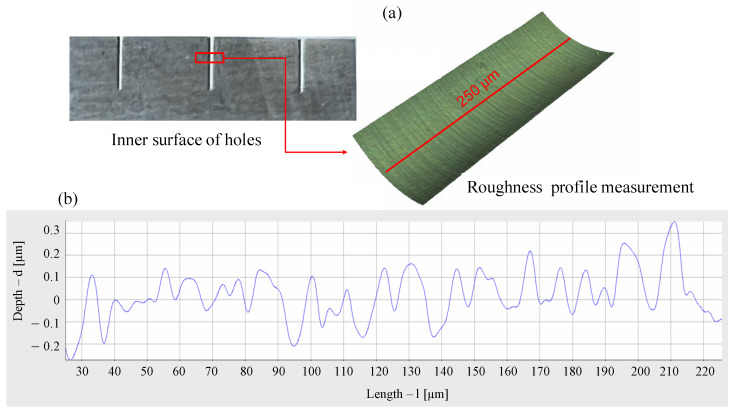
Roughness profile measurement ($D_{\mathrm{hole}}$ = 0.35 mm, $v_{c}$ = 25 m/min, $f_{z}$ = 5 μm). (**a**) Selected region for roughness measurement. (**b**) Roughness profile.

A second microdrilling operation was performed in order to measure the roughness of the inner surface of the holes as a relevant factor to assess the corrosion rate of Mg-based implants that is expected to increase at lower surface quality [31]. Therefore, as a biodegradable medical metallic material, the surface roughness of magnesium is very important for its service life. In this case, the microholes were drilled in the same run order with the same strategy as the previous tests (see Table 2, Table 3, Table 4 and Figure 4). The holes were arranged to create a square on the surface as shown in Figure 5b. To remove excess material and expose the surface of the holes, the cylindrical specimen was milled using a contour cycle. In this way, the mill passed along the sides of the square tangentially to the diameter of the holes so that they were not completely exposed. Then, with a 800 mesh SiC paper, the extra material was removed manually from the side faces of the specimens (see Figure 8a). Due to the fact that the contouring operation slightly damaged some areas of the inside of the hole, the inner surfaces were not captured in their full length but only in a defect-free central area with a maximum length of 300 μm with a 100× magnification. The average roughness profile Ra has been obtained by drawing a line along the axis of the hole with the Alicona software as shown in Figure 8a, which is a detail taken from Figure 5b. Therefore, the roughness profile lengths were kept equal to 250 μm for the 0.20 mm and 0.35 mm holes. The cut off wavelength was kept equal to 800 μm. An example of the obtained roughness profile is shown in Figure 8b.

## 3. Results

### 3.1. Chip Analysis

**Figure 9:**
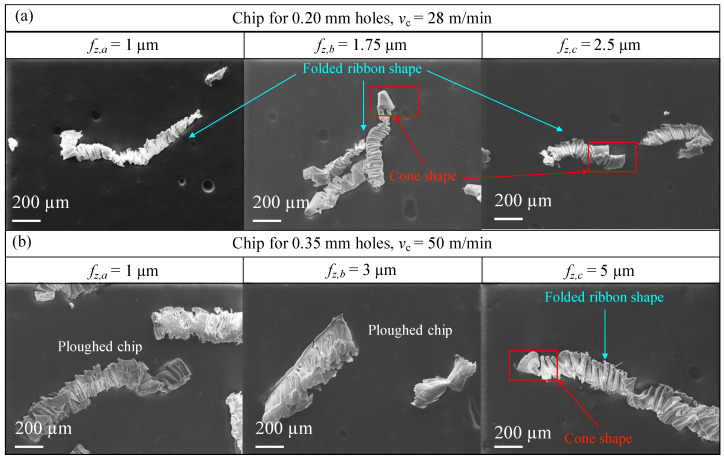
SEM images of the chip collected at different feed values. (**a**) Chip obtained with 0.20 mm microdrill. (**b**) Chip obtained with 0.35 mm microdrill.

The formed chip at varying $f_{z}$ is shown in Figure 9. For feeds equal to 2.5 and 5 μm, for the 0.20 mm and the 0.35 mm respectively, more or less long chips can be observed, which look almost crumpled and do not form a spiral structure. Specifically, two shapes can be distinguished: (a) transition between spiral cone and folded ribbon, and (b) folded ribbon. This phenomenon occurs because at first the chip should form a spiral shape as the tool is engaged with the material to be cut. Subsequently, the chip is deformed due to the increased resistance to chip expulsion as the depth of the hole increases. In this way the material folds into a shape called folded ribbon [29]. The ductility and low hardness of magnesium cause the chip inside the small flute to pack on itself as it moves upwards during drilling, which forms numerous folds (see Figure 9).

For the chips obtained from the 0.35 mm holes, flattened shapes are observed, as if the material had been ploughed. This effect results from the fact that the tool cutting edge radius of 4.52 μm (see Table 2) is much greater than the theoretical chip thicknesses, shown in Table 6. In the case of the chip obtained from the 0.20 mm holes, this effect seems less pronounced. In fact, the chip maintains a constant shape down to the 1 μm feed.

**Figure 10:**
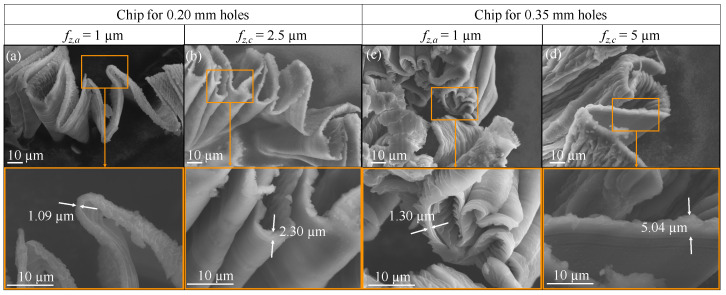
Chip thickness measurement from SEM images. (**a**) Chip obtained form the 0.20 mm holes and its detail for $f_{z}$ = 1 μm. (**b**) Chip thickness obtained form the 0.20 mm holes and its detail for $f_{z}$ = 2.5 μm. (**c**) Chip obtained form the 0.35 mm holes and its detail for $f_{z}$ = 1 μm. (**d**) Chip thickness obtained form the 0.35 mm holes and its detail for $f_{z}$ = 5 μm.

Chip thickness was measured with ImageJ from SEM images for chips obtained at 1 μm feed for 0.20 mm and 0.35 mm holes. The results are shown in Figure 10a,c. The values resulting from this analysis are higher than the theoretical values reported in Table 6 ($h_{0.20}$ = 0.87 μm and $h_{0.35}$ = 0.91 μm).

The same occurs with the measurement of chip thickness, for feeds of 2.5 μm and 5 μm for the 0.20 mm and 0.35 mm holes respectively (Figure 10b,d. In fact, in both cases the measured values of *h* are higher but very close to that calculated by means of the Equation (1) showed Table 6 ($h_{0.20}$ = 2.17 μm and $h_{0.35}$ = 4.53 μm). It can be confirmed that under these conditions, the removal of material takes place in the correct manner.

### 3.2. Hole Quality

**Figure 11:**
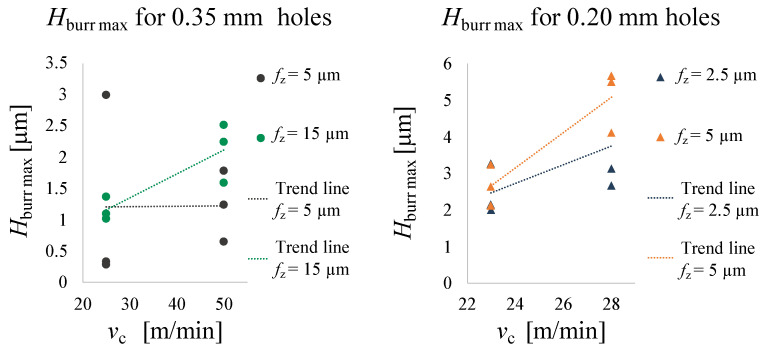
$H_{\mathrm{burr}\max}$ trend as a function of $v_{c}$.

**Figure 12:**
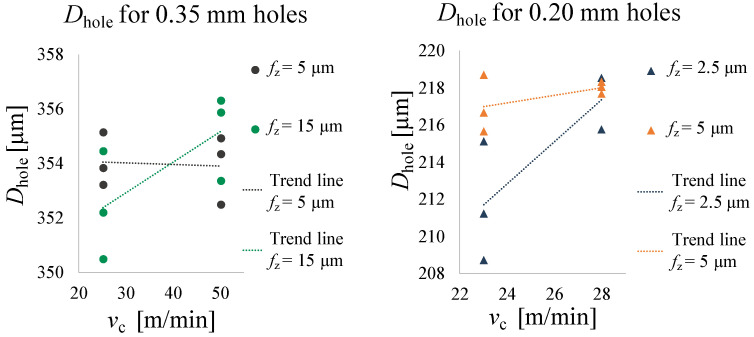
$D_{\mathrm{hole}}$ trend as a function of $v_{c}$.

**Figure 13:**
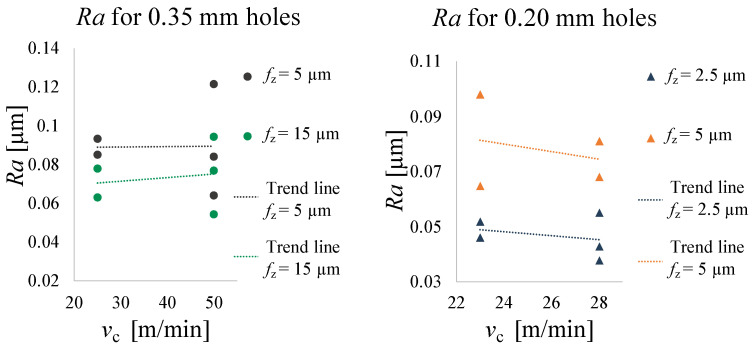
Ra trend as a function of $v_{c}$.

Normality and homoscedasticity of the residuals for burr height, diameter and roughness measurements were checked and no outliers were found. ANOVA tests were conducted using the data analysis software Minitab (Minitab, Ltd., Coventry, UK) to verify if $v_{c}$, $f_{z}$ or the interaction between $v_{c}$ and $f_{z}$ affect the quality of the microholes. A type I error = 5% was considered and factor interactions were included up to the 2nd order. Table 7 provides all the collected data for the hole quality analysis, also graphed in Figure 11, Figure 12 and Figure 13.

#### 3.2.1. Burr Height

Table 8 shows the output results of the ANOVA test for burr height and the value of $H_{\mathrm{burr}\max}$ as a function of $v_{c}$ is shown in Figure 11. The ANOVA test showed that $H_{\mathrm{burr}\max}$ is influenced by $v_{c}$ for the 0.20 mm microholes (*p*-Value = 0.011), but not by $f_{z}$ or their interaction $v_{c}$·$f_{z}$. In fact, the values of $H_{\mathrm{burr}\max}$ are higher for $v_{c}$ = 28 m/min at the same $f_{z}$. This phenomenon could be related to thermal softening of Mg caused by the increasing temperature due to the cutting speed. For the 0.35 mm holes $H_{\mathrm{burr}\max}$ results are not statistically different: the ANOVA tests showed that $v_{c}$, $f_{z}$ and $v_{c}$·$f_{z}$ do not influence the value of $H_{\mathrm{burr}\max}$. For both the 0.20 mm and 0.35 mm holes, the shape of the burrs is smooth and their heights are not uniform, but vary greatly around the circumference. This could be attributed to the high plastic deformation and ductility of Mg and the intrinsic variability of the process in the micrometric range.

#### 3.2.2. Diameters

Table 9 shows the output results of the ANOVA test for entrance diameters. This analysis showed that the $D_{\mathrm{hole}}$ of 0.20 mm holes are affected by both $v_{c}$ and $f_{z}$ (*p*-Value ($v_{c}$) = 0.018, *p*-Value ($f_{z}$) = 0.027), but not by their interaction. In particular, $D_{\mathrm{hole}}$ is higher for larger $v_{c}$ and $f_{z}$. No statistically differences are present for the 0.35 mm hole diameters, and no factor shows evidence of affecting the results. Nevertheless, it is observed that the measured diameters deviate from the nominal diameter of the tools (see Figure 12). In particular, the results show that $D_{\mathrm{hole}}$ is always larger than the nominal one. These outcomes are associated with the tool runout oscillation at high spindle speeds, which is not negligible in micromachining, even if it was measured as a total indicator reading (TIR) just 2 μm in air at the working revolution speed with the VTS presetter. Tool runout contributes to the increase of the effective tool diameter during its rotation, together with a tool buckling effect due to the thrust force.

#### 3.2.3. Roughness

The outputs of the statistical analysis reported in Table 10 shows that Ra for 0.20 mm holes depends only on $f_{z}$ (*p*-Value ($f_{z}$) = 0.047). In fact, as graphed in Figure 13, Ra increases by increasing $f_{z}$. There is no statistically significant difference for Ra of the 0.35 mm holes.

**Figure 14:**
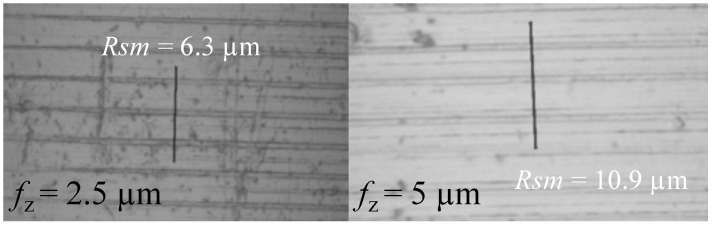
Rsm value for 0.20 mm holes. On the left, the 15 μm line drawn on the inner surface of the holes obtained with $f_{z}$ at 2.5 μm, on the right with 5 μm.

In addition, grooves were observed in the 0.2 mm holes. As shown in Figure 14 which is a detail taken from Figure 8a, the RoughnessProfileMeasurements was used to assess the distance between these grooves by drawing a 15 μm line. The mean spacing of profile irregularities of roughness profile *Rsm* was then considered. Its value is twice $f_{z}$. This phenomenon could be caused by a piece of hardened material stuck on a single cutting edge, as it occurs once per revolution.

## 4. Discussion

Micromechanical cutting methods are commonly used to obtain the final shape of the magnesium-based medical devices, but very few studies can be found in literature describing their objective and repeatable application [14,15]. Furthermore, deep-hole microdrilling is very challenging for pure magnesium, especially for high aspect ratios.

The work reported here was focused on testing different cutting conditions, with the purpose to understand the suitability of microdrilling for the future manufacture of a Mg-based intraocular drug delivery device for AMD treatment, or other Mg-based biomedical devices. This preliminary study was performed with 0.20 mm and 0.35 mm microdrills, using a full factorial design in which cutting speed $v_{c}$ and feed $f_{z}$ were varied over two levels. The use of pecks in this microdrilling process proved to be very advantageous for obtaining holes with a high $Q_{\mathrm{hole}}$/$D_{\mathrm{hole}}$.

The chip analysis confirmed how the chip removal process took place regularly producing a deformed chip thickness higher than the undeformed one, as usual, even if these cutting conditions should have been under the minimum chip thickness and so in a region where the chip should not have been properly formed. The reported results show how the chips are ploughed but it was still possible to form chips, suggesting that the minimum chip thickness could be lower than predicted. Moreover, for a feed per tooth equal to 1 μm, the drilling process is still capable to form a chip, even if it is not optimal. In fact, when $f_{z}$ is as low as 1 μm, the chip not only packs into folded structures, but in some cases appears ploughed. This phenomenon indicates that microdrilling must be performed for higher feed values, so as not to cause excessive tool wear or even tool breakage due to chip clogging. In any case, the simple fact that the chip is formed at 1 μm of feed per tooth seems to point out as the minimum chip thickness for pure magnesium is lower than expected, which means lower than the aluminium one. On the other hand, the measurement of chip thickness, for feeds of 2.5 μm and 5 μm for the 0.20 mm and 0.35 mm holes respectively, confirmed the correct choice of cutting parameters for the drilling, as the measured h is slightly higher than the uncut chip thickness.

Furthermore, in a proper microdrilling process, the chip should break up into short segments and not remain on the tool body [29]. This optimal condition is only achieved with higher feeds. As it is not possibile to achieve the feeds able to naturally break the chip in this study in order not to produce excessive forces, it was verified how the chip forms in the studied parameters range and then peck drilling strategy was selected for the chip breaking purpose.

The results of microdrilling experiments showed that the maximum burr height of magnesium is influenced by the cutting speed for the 0.20 mm holes. In fact, $H_{\mathrm{burr}\max}$ increases with higher cutting speeds, probably due to the thermal effect that plasticizes the material. Being an unavoidable process during drilling, the formation of burrs must be minimised: the removal of burrs involves similar issues respect to conventional drilling, but their small size makes them difficult to observe, measure and remove. In light of this, the results have proved that low cutting speed values result in lower burr height and thus better micro-hole quality for magnesium. In addition, this working parameter also affects the entrance diameter. This can probably be attributed to the runout of the tool, which may increase due to the high speeds, thus increasing the size of the flying diameter of the tool entering the hole. For this reason it is necessary to work at low cutting speeds during prototype development to remain within tolerances.

Furthermore, the outcomes related to the 0.20 mm holes showed that the cutting parameter $f_{z}$ determines the quality of the hole in terms of entry diameter and internal roughness. These results are in agreement with the literature. As reported in the state of the art, the feed is the most influential parameter in mechanical cutting methods. As the feed increases, the cutting forces increase [15,32]. In the present work, hole entrance diameters are larger than the nominal tool diameters, and their values increase as $f_{z}$ increases. This phenomenon can therefore lead to greater deformation of the tool for the buckling effect, which in turn causes greater tool diameters. The same happens for Ra of the inner surface of the holes, which, as is common knowledge, assumes higher values with increasing $f_{z}$ for purely geometric causes.

In addition, a study about the influence of the material microstructure on the machining characteristics of copper in microturning showed that when the feed is equal to the grain size, the roughness of the machined surface is lower [33]. The results obtained in the present study about microdrilling, in fact, show that the roughness Ra for 0.20 mm holes is lower for a $f_{z}$ = 2.5 μm (closer to grain size $\bar{d}$ = 3 μm than for 5 μm), as the cutting occurs largely at the grain boundaries.

Nevertheless, the 0.35 mm drilling tests did not show any relevant results on the effect of process parameters. This could be attributed to the fact that a wider range of $v_{c}$ and $f_{z}$ should be used to observe significant differences.

## 5. Conclusions

The presented approach is effective to drill magnesium with high depth-to-diameter ratios, which shows that the selected technology is suitable to be used in the future for prototyping the intraocular drug delivery device and other Mg-based biomedical devices. In particular, the relevant outcomes of this work are mentioned below.

- Comparison of the theoretical and measured chip thickness showed that the cutting parameters selected for the experiment allow machining to be carried out under cutting and not ploughing conditions.
- Chip formation with $f_{z}$ = 1 μm demonstrated that the minimum chip thickness for magnesium may be less than expected, and therefore less than the aluminium one.
- The feed $f_{z}$ has an influence on both the entrance diameters and the roughness of the internal surfaces of the hole. In fact, the value of hole diameters and the parameter Ra increase as $f_{z}$ increases. This means that it is necessary to work at low feeds, both to obtain entry diameters closer to the nominal tool diameter and to achieve low roughness values.
- The cutting speed $v_{c}$, on the other hand, influences the formation of burrs during magnesium microdrilling and affects the final entrance diameter of the hole. Their values increases as the $v_{c}$ increases. For this reason, in order to achieve better hole quality, it is necessary to work at low cutting speeds.

Magnesium manufacturing is a very interesting topic in the biomedical field, considering how this material is an excellent candidate for the development of devices that must meet biocompatibility and biodegradability requirements. This study opens up other avenues for research. Characterizing the tool wear, measuring and modelling the involved cutting forces to have a better insight of the process and prototyping a miniaturized device are among the areas of future and current development.

## Figures and Tables

**Table 1:** Sample properties.

Hardness	Density
52.6 HV	1.73 g/cm^3^

**Table 2:** Microtwist drills properties. *D*_tool_: tool diameter; *L*_flute_: flute length; *L_u_*: tool usable length; ϵ: point angle; *r_e_*: cutting tool radius; Coating: commercial name of the coating.

TOOL ID	*D*_tool_ [mm]	*L*_flute_ [mm]	*L_u_* [mm]	ϵ [${}^{\circ }$]	*r_e_* [μm]	Coating
Custom	0.20	2	4	120	2.95	Solo
2.CD.080035.IN	0.35	2.8	2.8	130	4.52	eXedur RIP

**Table 3:** Cutting conditions (hole geometry). *D*_hole_: hole diameter; *Q*_pilot_: pilot depth; *Peck*_i-th_: i-th peck depth; *Q*_hole_: blind hole depth; *Q*_hole_/*D*_hole_: aspect ratio.

*D*_hole_ [mm]	*Q*_pilot_ [mm]	*Peck*_i-th_ [μm]	*Q*_hole_ [mm]	*Q*_hole_/*D*_hole_
0.20	0.67	65	3.9	20
0.35	0.9	175	2.5	7

**Table 4:** DoE scheme.

**Factors (0.20 mm)**	**Level 1 (−)**	**Level 2 (+)**
$v_{c}$ [m/min]	23	28
$f_{z}$ [μm]	2.5	5
**Factors (0.35 mm)**	**Level 1 (−)**	**Level 2 (+)**
$v_{c}$ [m/min]	25	50
$f_{z}$ [μm]	5	15

**Table 5:** Drilling conditions used for the chip formation analysis.

*D*_tool_ [mm]	$v_{c}$ [m/min]	$f_{z,a}$ [μm]	$f_{z,b}$ [μm]	$f_{z,c}$ [μm]
0.20	28	1	1.75	2.5
0.35	50	1	3	5

**Table 6:** Theoretical values of chip thickness under the considered conditions.

***D*_tool_ = 0.20 mm**			
**$f_{z}$** [μm]	1	1.75	2.5
***h*** [μm]	0.87	1.52	2.17
*****D*****_tool_** = 0.35 mm**			
**$f_{z}$** [μm]	1	3	5
***h*** [μm]	0.91	2.72	4.53

**Table 7:** Observations obtained from 0.20 mm and 0.35 mm diameter holes at different cutting conditions. As the surfaces were damaged by the contouring operation, it was not possible to measure *Ra* for holes 2 and 8 with 0.20 mm diameters and holes 4 and 11 with 0.35 mm diameters.

	0.20 mm Holes					0.35 mm Holes				
Run Order	$v_{c}$ [m/min]	$f_{z}$ [μm]	$H_{\mathrm{burr}\max}$ [μm]	$D_{\mathrm{hole}}$ [μm]	Ra [μm]	$v_{c}$ [m/min]	$f_{z}$ [μm]	$H_{\mathrm{burr}\max}$ [μm]	$D_{\mathrm{hole}}$ [μm]	Ra [μm]
1	23	2.5	2.01	215.15	0.052	50	15	2.24	353.37	0.094
2	23	2.5	2.16	208.71	-	50	5	1.24	352.50	0.084
3	28	5	4.10	217.70	0.050	25	5	2.99	355.14	0.093
4	28	2.5	3.13	217.70	0.038	25	15	1.02	350.48	-
5	28	2.5	2.66	215.80	0.043	25	5	0.29	353.20	0.085
6	23	5	3.24	215.69	0.068	25	15	1.10	352.20	0.063
7	23	2.5	3.27	211.21	0.046	50	5	1.78	354.35	0.064
8	23	5	2.13	218.72	-	50	15	1.59	356.31	0.054
9	23	5	2.64	216.67	0.065	25	15	1.37	354.45	0.078
10	28	5	5.50	218.09	0.098	50	15	2.51	355.87	0.077
11	28	2.5	5.50	218.56	0.055	25	5	0.33	353.82	-
12	28	5	5.66	218.32	0.081	50	5	0.66	354.92	0.121

**Table 8:** ANOVA table for $H_{\mathrm{burr}\max}$: Minitab software outputs. DF = Total Degrees of Freedom; Adj SS = Adjusted Sum of Squares; Adj MS = Adjusted Mean Squares.

		0.20 mm Holes				0.35 mm Holes			
Source	DF	Adj SS	Adj MS	F-Value	*p*-Value	Adj SS	Adj MS	F-Value	*p*-Value
$v_{c}$	1	10.2675	10.2675	10.71	0.011	0.7105	0.7105	0.96	0.356
$f_{z}$	1	1.7176	1.7176	1.79	0.218	0.5376	0.5376	0.73	0.419
$v_{c}$·$f_{z}$	1	0.9633	0.9633	1.00	0.346	0.6440	0.6440	0.87	0.379
Error	8	7.6723	0.9590			5.9311	0.7414		
Total	11	20.6208				7.8233			

**Table 9:** ANOVA table for $D_{\mathrm{hole}}$: Minitab software outputs. DF = Total Degrees of Freedom; Adj SS = Adjusted Sum of Squares; Adj MS = Adjusted Mean Squares.

		0.20 mm Holes				0.35 mm Holes			
Source	DF	Adj SS	Adj MS	F-Value	*p*-Value	Adj SS	Adj MS	F-Value	*p*-Value
$v_{c}$	1	33.40	33.40	8.89	0.018	5.3734	5.3734	2.37	0.162
$f_{z}$	1	27.18	27.18	7.24	0.027	0.1302	0.1302	0.06	0.817
$v_{c}$·$f_{z}$	1	16.24	16.24	4.32	0.071	6.4680	6.4680	2.86	0.130
Error	8	30.05	3.756			18.1211	2.2651		
Total	11	106.87				30.0927			

**Table 10:** ANOVA table for Ra: Minitab software outputs. DF = Total Degrees of Freedom; Adj SS = Adjusted Sum of Squares; Adj MS = Adjusted Mean Squares.

		0.20 mm Holes				0.35 mm Holes			
Source	DF	Adj SS	Adj MS	F-Value	*p*-Value	Adj SS	Adj MS	F-Value	*p*-Value
$v_{c}$	1	0.000023	0.000023	0.1	0.762	0.000016	0.000016	0.04	0.855
$f_{z}$	1	0.001411	0.001411	6.23	0.047	0.000660	0.000660	1.51	0.265
$v_{c}$·$f_{z}$	1	0.000109	0.000109	0.48	0.513	0.000009	0.000009	0.02	0.892
Error	6	0.001360	0.000227			0.002623	0.000437		
Total	9	0.003130				0.003304			

## Data Availability

Not applicable.
